# Reliability of respiratory pressure measurements in ventilated and non-ventilated patients in ICU: an observational study

**DOI:** 10.1186/s13613-018-0362-1

**Published:** 2018-01-30

**Authors:** Clément Medrinal, Guillaume Prieur, Yann Combret, Aurora Robledo Quesada, Tristan Bonnevie, Francis Edouard Gravier, Eric Frenoy, Olivier Contal, Bouchra Lamia

**Affiliations:** 1 Normandie Univ, UNIROUEN, EA3830 - GRHV, 76000 Rouen, France; 2 Institute for Research and Innovation in Biomedicine (IRIB), 76000 Rouen, France; 3 Intensive Care Unit Department, Groupe Hospitalier du Havre, Avenue Pierre Mendes France, 76290 Montivilliers, France; 4 Pulmonology Department, Groupe Hospitalier du Havre, Avenue Pierre Mendes France, 76290 Montivilliers, France; 5 Institut de Recherche Expérimentale et Clinique (IREC), Pôle de Pneumologie, ORL and Dermatologie, Université Catholique de Louvain, Brussels, 1200 Belgium; 6 Physiotherapy Department, Groupe Hospitalier du Havre, Avenue Pierre Mendes France, 76290 Montivilliers, France; 7 ADIR Association, Bois Guillaume, France; 8 Intensive Care Unit Department, Hôpital Jacques Monod, 76290 Montivilliers, France; 9 University of Applied Sciences and Arts Western Switzerland (HES-SO), Avenue de Beaumont, 1011 Lausanne, Switzerland; 10 Intensive Care Unit, Respiratory Department, Rouen University Hospital, Rouen, France

**Keywords:** Intensive care unit, Mechanical ventilation, Respiratory muscles

## Abstract

**Background:**

Assessment of maximum respiratory pressures is a common practice in intensive care because it can predict the success of weaning from ventilation. However, the reliability of measurements through an intubation catheter has not been compared with standard measurements. The aim of this study was to compare maximum respiratory pressures measured through an intubation catheter with the same measurements using a standard mouthpiece in extubated patients.

**Methods:**

A prospective observational study was carried out in adults who had been under ventilation for at least 24 h and for whom extubation was planned. Maximal respiratory pressure measurements were carried out before and 24 h following extubation.

**Results:**

Ninety patients were included in the analyses (median age: 61.5 years, median SAPS2 score: 42.5 and median duration of ventilation: 7 days). Maximum respiratory pressures measured through the intubation catheter were as reliable as measurements through a standard mouthpiece (difference in maximal inspiratory pressure: mean bias = − 2.43 ± 14.43 cmH_2_O and difference in maximal expiratory pressure: mean bias = 1.54 ± 23.2 cmH_2_O).

**Conclusion:**

Maximum respiratory pressures measured through an intubation catheter were reliable and similar to standard measures.

Clinical trial registration Retrospectively Registered in ClinicalTrials.gov (NCT02363231).

## Background

Mechanical ventilation generally results in a loss of respiratory muscle strength [1, 2]. The prevalence of respiratory muscle weakness is high, and the causes are multifactorial [3–5]. Assessment of respiratory muscle strength is becoming common practice in intensive care. Assessment techniques range from diaphragm ultrasound to measurement of maximum respiratory pressures. Respiratory muscle strength has been established as prognostic of successful weaning and mortality [6–8]. Measurement of maximum respiratory pressures is a simple, non-invasive method to quantify the global strength of the inspiratory and expiratory muscles. Pressures can be measured using a manometer with a unidirectional valve or the “Negative Inspiratory Force” (NIF) function available on most ventilators. However, these methods require full patient cooperation. Several protocols have thus been developed for use in intensive care to ensure accurate measurements with or without cooperation from the patient [9]. Several studies have attempted to determine optimal methods to ensure quality measurements that are reliable [10–12].

Respiratory pressure measurements are commonly carried out, while the patient is intubated as part of the evaluation to determine the likely success of extubation [5, 7]. It is important to carry out longitudinal evaluations of respiratory muscle strength after mechanical ventilation in order to increase understanding of the relationship between strength and long-term rates of mortality [7]. However, the methods used to measure respiratory pressures differ between intubation and extubation and, along with other factors such as lack of patient cooperation and discomfort, this could lead to different values being recorded. To our knowledge, no, or few, studies have evaluated respiratory pressure measurements in non-ventilated patients in ICU, and the reliability of these measurements has not been compared between intubation and extubation.

The aim of this study was to compare maximum respiratory pressures measured through an intubation catheter (intubated patients) with the same measurement using a standard mouthpiece (extubated patients). The secondary aims were to analyse correlations between the two measurements.

## Method

### Study design and participants

This study was part of a larger, prospective observational cohort study conducted in an 18-bed intensive care unit (ICU) between January 2014 and December 2014 [7]. The study was approved by our Institutional Review Board (Comité de Protection des Personnes Nord-Ouest 3); NCT02363231 www.clinicaltrials.gov. In conformity with the Declaration of Helsinki, all patients participated voluntarily.

Patients were included if they were over 18 years of age and had undergone a minimum of 24 h of MV. They were not included if they had chronic loss of autonomy (a KATZ score below 6/6 [13], a degenerative neurological pathology with disabling muscle weakness, were agitated prior to the evaluation (Ramsay score of 1 or Richmond Agitation-Sedation Scale (RASS) greater than 1) or a decision to withhold life sustaining treatment had been made. Patients who were included but had to be re-intubated during the first 24 h of extubation were excluded from the analysis.

### Study protocol

In our ICU, patients are assessed daily (without sedation) to determine whether they are ready to wean from MV. If a patient fulfils extubation criteria and level of cooperation is satisfactory, a weaning trial is carried out under pressure support (inspiratory positive airway pressure of 7 cmH_2_O with no expiratory positive airway pressure for 30–120 min) [14]. For the purpose of the study, if the trial was successful and extubation was planned, the patient underwent maximum inspiratory and expiratory pressure measurements (MIPs and MEPs) (intubation condition). Twenty-four hours following extubation, MIPs and MEPs were re-measured, this time using a mouthpiece (mouthpiece condition).

Demographic data, reasons for admission to ICU and comorbidities were collected at the time of inclusion, prior to carrying out the MIP and MEP measurements under MV.

In both conditions, the MIP and MEP measurements were carried out with the patient lying in bed with the backrest inclined to 45°. Respiratory physiotherapy was carried out first to ensure that secretions were evacuated, and endotracheal aspiration was carried out for intubated patients.

An electronic manometer, micro-RPM^®^ (Eolys, PAYS), with a unidirectional valve was used to measure respiratory pressures. In both conditions, MIP was measured at the residual volume and patients were instructed accordingly.

In the intubation condition, the manometer was connected to the endotracheal tube using a catheter mount. The patient was disconnected from the ventilator for a minimum of 20 s [11].

In the mouthpiece condition, it was not possible to leave the manometer in position for 20 s. MIP was measured after a maximal exhalation (at the residual volume).

MEP was measured after a maximal inspiration in both conditions. Three MIP and three MEP measurements were carried out for each patient, and the best result was used for the analysis.

### Statistical analysis

Descriptive statistics are reported as counts and percentages for categorical data, and means and standard deviations or medians and 25th–75th percentiles for continuous variables, depending on the normality of the distribution. Differences between values were evaluated using a Wilcoxon matched-pairs signed rank test. Univariate linear regression analysis was performed using the least squares method. The Bland–Altman limits of agreement method was used to calculate bias and precision.

Statistical analyses were performed using GraphPad Prism 5. A two-tailed *p* value of 0.05 was considered significant for all analyses.

## Results

One hundred and twenty-four patients were included in the larger study. Of these, 101 accepted to carry out additional measurements. Eleven patients required re-intubation within 24 h of extubation and were excluded from the analysis. Ninety patients thus underwent MIP and MEP measurements in both conditions.

Patient characteristics are described in Table 1. Briefly, 43% of the patients were women, median age was 61.5 years, median BMI was 28.6 kg/m^2^, median SAPS2 score was 42.5 and median duration of MV was 7 days.

**Table 1:** Cohort characteristics

	*N* = 90
Female, *n* (%)	39 (43)
Age, mean (SD)	61.5 (14)
Body mass index (Kg/m^2^), median (25th–75th percentile)	28.6 (24.4–32)
SAPS II at ICU admission, median (25th–75th percentile)	42.5 (31–57)
No. of admissions to ICU within the last year, *n* (%)	4 (4.4)
*Main diagnosis*	
Pneumonia, *n* (%)	32 (35)
Sepsis, *n* (%)	8 (9)
COPD/asthma exacerbation, *n* (%)	12 (13)
Cardiac failure, *n* (%)	12 (13)
Drug overdose/acute mental status change, *n* (%)	11 (12)
Intra-abdominal sepsis with surgery, *n* (%)	14 (15)
Trauma, *n* (%)	1 (4)
*Co*-*morbidity*	
Chronic pulmonary disease, *n* (%)	23 (25)
Obesity, *n* (%)	27 (30)
Chronic cardiac insufficiency, *n* (%)	13 (14)
Cancer, *n* (%)	15 (17)
Chronic kidney disease, *n* (%)	14 (15)
Diabetes mellitus, *n* (%)	17 (19)
*Between admission and awakening*	
Septic shock, *n* (%)	45 (50)
ARDS, *n* (%)	13 (14)
Renal failure, *n* (%)	30 (33)
Use of catecholamines, *n* (%)	58 (64)
Use of neuromuscular blockers, *n* (%)	58 (64)
No. of days of neuromuscular blockers, median (25–75th percentile)	1 (0–3)
Use of corticosteroids, *n* (%)	21 (78)
Ventilator use (days), median (25th–75th percentile)	7 (4–9)

*SAPS* simplified acute physiology score, *ICU* intensive care unit, *No.* number, *COPD* chronic obstructive pulmonary disease, *ARDS* acute respiratory distress syndrome

Median MIP value was 28 (21.7–40.2) cmH_2_O in the intubation condition and 27 (19–38) cmH_2_O in the mouthpiece condition (*p* = 0.02). Linear regression showed a significant correlation between the values in each condition (*r* = 0.64 95% CI [0.5–0.75]; *p* < 0.0001).

**Fig. 1:**
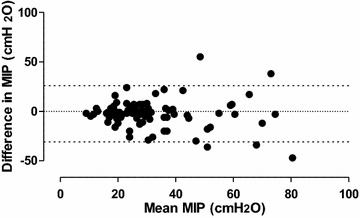
Bland–Altman analysis of maximal inspiratory pressure correlations: difference versus mean

The Bland–Altman analysis showed that the MIP values between intubation and extubation were clinically comparable (mean bias (ΔMIP) = − 2.43 ± 14.43 cmH_2_O). (See Fig. 1).

There was no statistically significant difference in MEP values between conditions [47 (30–74) vs. 53.5 (34–76.2) cmH_2_O; *p* = 0.2]. There was a strong significant correlation between the MEP values in each condition (*r* = 0.71 95% CI [0.6–0.8]; *p* < 0.0001).

**Fig. 2:**
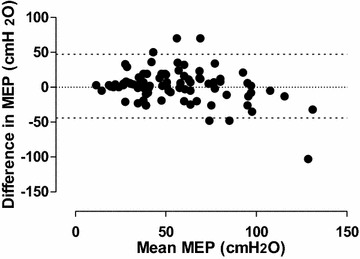
Bland–Altman analysis of maximal expiratory pressure correlations: difference versus mean

There was no clinical difference between the values in the two conditions as shown by the Bland–Altman analysis (mean bias (ΔMEP) = 1.54 ± 23.2 cmH_2_O) (See Fig. 2).

No patient-related factors were found to be associated with the measurement bias (age, BMI, SAPS2, number of days under mechanical ventilation, extubation failure). However, there was a correlation between the ΔMIP and the ΔMEP (*r* = 0.49 95% CI [0.31–0.64]; *p* < 0.0001).

There was a significant correlation between MIP and MEP values in each condition (respectively *r* = 0.61 95% CI [0.45–0.72]; *p* < 0.0001 and *r* = 0.66 95% CI [0.52–0.77]; *p* < 0.0001).

## Discussion

This study found [1] that the methods of measuring respiratory pressures in intubated and extubated patients produced clinically similar results for both MIP and MEP, and [2] there were strong correlations between the MIP and MEP values in both conditions.

Assessment of respiratory pressures is common practice in ICU [4, 5, 7, 9–12]. Although other tools may more accurately assess muscle strength, measures of respiratory pressure are used to determine if a patient is ready to wean from MV, as well as the prognosis [7, 15]. For this reason, we believed it was important to evaluate the validity of measurements in intubated patients compared with post-extubation measurements using a mouthpiece in order to longitudinally evaluate changes in respiratory muscle strength.

Measurement of maximal respiratory pressures requires patient cooperation, which can be difficult when patients are intubated; however, similar pressures were recorded during intubation and extubation, with slightly higher pressures during intubation. This could be explained by the fact that mouth leak cannot occur when the patient is intubated with the balloon inflated or because the measurement was carried out over 20 s when the patients were intubated [11]. One study compared the conventional method (values taken at the maximum pressure plateau maintained for at least 1 s) with Marini’s method [10] (measurement of inspiratory pressure with a unidirectional valve over 20 s) in 54 patients. MIP was 28% higher using Marini’s method with a coefficient of variation of around 10%, indicating good reliability. This procedure can be used for intubated patients but is not reliable in extubated patients. Nevertheless, in the present study, mean MIP variation between the two conditions was − 2.43 cmH_2_O (− 8.4%) and for MEP was 1.54 cmH_2_O (7%), confirming good reliability across conditions and measurements.

The results of this study showed a relationship between MIP and MEP. MEP reflects the patient’s capacity to cough, and a low MEP is associated with delayed weaning [15]; however, studies tend to focus on inspiratory muscle strength, neglecting expiratory muscle strength. MIP is reported to be predictive of successful extubation, and we recently showed that low MIP before extubation (MIP ≤ 30 cmH_2_O) was an independent predictor of an increase in mortality risk 1 year following extubation [7]. However, several authors have stated that values obtained in intubated patients may be underestimated [9, 12, 15]. In the current study, we found that Marini’s method (occlusion for 20 s) produced clinically similar values to measurements carried out with a mouthpiece following recommendations [16]. This indicates that if the patient is sufficiently alert, the values are not underestimated and are therefore reliable across different conditions, allowing accurate follow-up of respiratory capacity.

This study has several limitations. Firstly, the observational design comprises several types of inherent bias and we did not perform a sample size calculation. Secondly, it was not possible to evaluate patients who were re-intubated within 24 h. Thirdly, the pressure measurements were not taken in exactly the same conditions. The second measurement 24 h following extubation may have been affected by respiratory muscle fatigue. Finally, we evaluated peak pressure, not pressure maintained over 1 s as recommended [16]. However, the recommendations are more relevant out of ICU where measurements of respiratory pressure differ considerably from the bedside measurements used in ICU [11].

This study has several strengths. The sample size was large and representative of the population of patients in ICU. The test evaluated is simple and easy to carry out at the patient’s bedside. Moreover, we showed that the measurements were reliable across two common conditions in ICU (intubated and extubated patients).

## Conclusion

Respiratory pressure measurements are reliable in both intubated and non-intubated patients. These results corroborate those of previous studies. Measurements of respiratory pressure can thus be carried out reliably when the patient is intubated and repeated following weaning from MV to carry out longitudinal evaluations of respiratory muscle recovery.

## Abbreviations

BMI: body mass index
CI: confidence intervals
ICU: intensive care unit
MIP: maximal inspiratory pressure
MEP: maximal expiratory pressure
MV: mechanical ventilation
NIF: negative inspiratory force
RASS: Richmond Agitation-Sedation Scale
SAPS: simplified acute physiology score
